# Attenuation of higher-order acoustic modes in a cylindrical waveguide using lined panel-cavity coupling

**DOI:** 10.1371/journal.pone.0345221

**Published:** 2026-04-22

**Authors:** Abdulwahed Alrashdi, Abdul Wahab, Aqsa Yaseen, Naif Alkuhayli, Hani Alahmadi

**Affiliations:** 1 Department of Mathematics, College of Science, Jouf University, Sakaka, Saudi Arabia; 2 Department of Mathematics, College of Science, Sultan Qaboos University, Muscat, Oman; 3 Department of Mathematics, COMSATS University Islamabad, Islamabad, Pakistan; Beni-Suef University, EGYPT

## Abstract

This work presents a mode-matching framework for analyzing acoustic attenuation in a waveguide that incorporates a centrally lined chamber, membrane discs at the interfaces, and extended inlet–outlet sections. The formulation also covers configurations with a lined cavity backed by rigid or soft discs, coupled to a single membrane disc at an interface adjoining an extended radiating region. The acoustic field within the waveguide is expressed through eigenfunction expansions, while the membrane response is modeled using a Galerkin procedure. The membrane displacement is projected onto orthogonal modal solutions constructed via Fourier series expansion. Applying interface continuity and orthogonality relations leads to truncated linear algebraic systems, which are solved numerically. These truncated solutions are then used to reconstruct matching conditions and confirm power conservation, thereby ensuring consistency of the formulation and convergence of modal amplitudes. The analysis reveals strong coupling between incident duct modes and localized cavity resonances, producing a panel–cavity interaction mechanism that drives selective attenuation. Numerical results highlight the efficiency of the proposed configuration in improving sound suppression over targeted frequency ranges under various lining conditions. The study offers practical insights for computational design and optimization of advanced noise-control solutions in ducted systems.

## 1 Introduction

Industrial noise control has become a major focus of research due to its significant impact on structural design, automotive and aviation industries, and heating, ventilation, and air conditioning (HVAC) systems. Noise and vibrations in mechanical systems arise from diverse and complex mechanisms. For instance, noise in automotive and aircraft systems primarily results from engine operations and airflow, while HVAC systems and industrial machinery generate noise through airflow and mechanical components. Effective noise control requires a thorough understanding of how energy propagates and distributes within physical systems, which is essential for improving noise reduction strategies and optimizing system performance.

Duct-like structures are prevalent in these settings, and their geometric and material properties have been widely studied [1–6]. A common noise mitigation approach involves the use of sound-absorbent linings to reduce acoustic energy transmission. Researchers have explored various strategies to enhance the performance of these linings. One approach involves designing materials with tailored impedance values to maximize acoustic attenuation within specific frequency ranges. Another strategy focuses on modifying structural properties along the acoustic impedance within guiding channels to improve noise reduction [7–9].

Elastic membrane components have also been explored for low-frequency noise attenuation [10]. The mode-matching method has been applied to analyze the fluid-structure responses of elastic plates under various lined conditions in flanged waveguides [11]. Lawrie and Afzal [12] investigated elastic membranes at the interface of two membrane-bounded duct regions of different heights, using tailored-Galerkin and Galerkin approaches to model the vibrational response of the bridging membrane. Additionally, varying lined conditions can significantly influence the performance of silencing devices. Locally reacting lined conditions, as opposed to bulk reacting lined conditions, have been shown to enhance sound attenuation performance [13]. For example, Du et al. [14]– [15] explored how lined conditions at membrane edges affect attenuation performance in a rectangular waveguide.

Panel-cavity coupling has been a subject of interest in previous studies. Pan and Bies [16] proposed an active noise reduction system based on an elastic membrane within a panel-cavity configuration. Kim and Brennan [17] introduced a design using an elastic plate with panel chambers to reduce sound from plane pistons and point sources. Ming and Pan [18] examined the sound insertion loss of acoustic enclosures with fluid-structure interaction under different boundary constraints. These studies relied on coupled mode theory, where initial solutions model the flexible segments’ response, and coupling coefficients are subsequently determined to describe acoustic propagation.

Coupled mode theory has limitations when dealing with strong coupling conditions or when continuity at the interfaces is not satisfied [19–22]. Alternative methods have been developed to address these issues. The Rayleigh-Ritz method has been used to solve boundary value problems involving elastic plates and rigid cavity interfaces [23,24]. Lawrie and Abrahams [25] proposed a mode-matching technique to analyze acoustic scattering in flexible ducts, which involved expanding duct region eigenfunctions and applying generalized orthogonality conditions [11,26–28].

This study presents a novel approach to optimizing acoustic attenuation in a targeted frequency range by modeling and analyzing a waveguide equipped with elastic components at the boundaries of a sound-absorbent lining cavity. The proposed method involves coupling incoming duct modes with flexible boundary components and localized modes within the lined cavity, forming a coupled panel-cavity system. This configuration enhances noise attenuation by leveraging the interaction between structural vibrations and acoustic propagation within the waveguide. The acoustic impedance of the lining is determined based on sound propagation in a locally reactive honeycomb layer supported by a rigid-wall boundary condition, resulting in a mixed-type boundary value problem. Recent contributions have highlighted how surface and interface effects, imperfect bonding, and multi-field couplings can strongly modify guided-wave dispersion, mode conversion, and energy transmission in smart and layered media. In particular, shear-horizontal (SH) and Love-type waves in piezoelectric, piezomagnetic and quasicrystalline settings have been analyzed under a variety of imperfect-interface models and surface theories, including higher-mode and topological features, flexoelectricity, and rheological/thermal-relaxation effects, with implications for sensing and wave-based diagnostics [29–37]

Mode-matching and membrane/liner elements have each been studied previously; however, the contribution here is their unified semi-analytical coupling in a single cylindrical framework: a centrally lined chamber modeled by a locally reacting impedance boundary condition, together with two elastic membrane interfaces at *z* = ±*L*. Recent studies have further extended mode-matching and hybrid semi-analytical strategies to treat wave-bearing cavities, finite lining segments, and coaxial lined chambers with flexible interfaces. These developments provide additional context for the present configuration, where a centrally lined chamber is coupled to two membrane junctions within a single cylindrical scattering framework [38–40]. The acoustic field is treated by mode matching, while the membrane boundary-value problem (with prescribed/clamped edge conditions and, more generally, mixed disk/annulus partitions) is handled efficiently via a Galerkin projection onto admissible basis functions [12], yielding a low-dimensional and well-conditioned algebraic representation that couples naturally to the acoustic modal amplitudes. The resulting formulation retains higher-order guided modes beyond plane-wave regimes, allows circumferentially varying impedance in the lined section, and accounts for fluid–structure interaction through both junction membranes within a single scattering model. The scheme is validated through reconstruction of the interface conditions and energy-balance checks, and it enables systematic comparison of rigid/soft/reactive and dissipative impedance cases within the same device setting, including transmission-loss predictions.

The structure of the article is as follows: Section 2 presents the mathematical formulation, including the acoustic and membrane disc responses using the Galerkin formulation. Section 3 describes the mode-matching solution under both symmetric and anti-symmetric configurations. The convergence of the proposed numerical technique is discussed in Section 4. Numerical results and conclusions are provided in Sections 5 and 6, respectively.

## 2 Problem formulation

**Fig 1:**
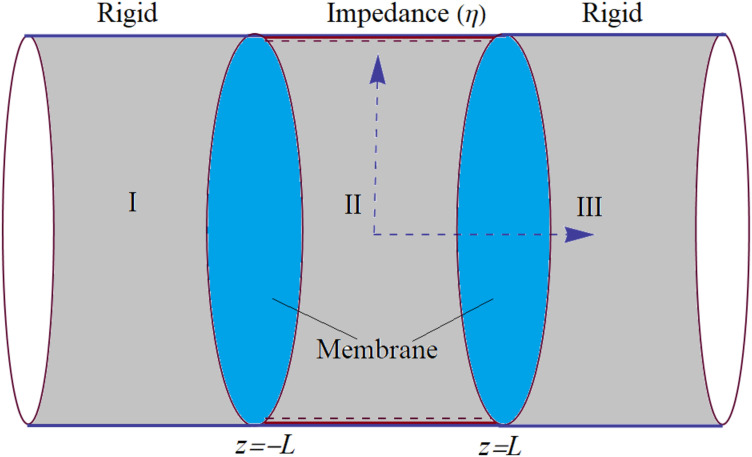
Physical configuration of the cylindrical waveguide.

Consider the wave propagation in a cylindrical waveguide containing elastic membranes at the interfaces of a chamber which is assumed to be bounded by rigid, soft, impedance, or sound absorbent type surfaces (see Fig 1). The interfaces are lying at $\bar{z}=\pm \bar{L}$ in the dimensional coordinate setting $\left(\bar{r},\bar{z}\right)$ and chamber is assumed by the region $|\bar{z}|<\bar{L}$, $\bar{0}\leq \bar{r}\leq \bar{a}$. Here the quantities with bars express the dimensional radial $\left(\bar{r}\right)$ and z-coordinate $\left(\bar{z}\right)$. The inside of the waveguide contains a compressible fluid of density ρ and sound speed *c*. The regions lying at $|\bar{z}|>\bar{L}$ are assumed as the extended inlet and outlet of the channel and these are bounded by the rigid wall conditions. The circular membranes lying at the interfaces are assumed to be fixed with a circular rim of radius $\bar{a}$. The dimensional equation of motion is given by [41]


$$T\bar{\nabla^{2}}\bar{𝒬}+\omega^{2}\rho_{m}\bar{𝒬}=[𝒫{]}_{-}^{+},$$ (1)

where $\bar{𝒬}$ represents the dimensional membrane displacement and $[𝒫{]}_{-}^{+}$ expresses the pressure difference across a membrane. Here quantities *T* and $\rho_{m}$ represent the tension and density of membrane disc repectively. Note that the harmonic time dependence $\exp(-i\omega \bar{t})$ is assumed and is suppressed throughout the article, while ω=ck is the radian frequency and *k* is the wavenumber. The rigid, soft, impedance, or sound absorbent surfaces can be defined by considering the impedance at the bounding surface. The impedance condition that describes the surfaces of extended inlet/outlet and chamber region is [12],


$$\bar{𝒫}-\bar{Z}\bar{\text{V}}\cdot \bar{\text{n}}=0,$$ (2)

where the vector quantities $\bar{\text{V}}=\bar{▽}\bar{\Phi }$ and $\bar{\text{n}}$ denote the velocity vector and unit normal vector directed into the surface, respectively. Here the counter $\bar{Z}$ expresses the dimensional form of the surface impedance. For $\bar{Z}\to \infty$ or 0, the surface behaves as rigid or soft, respectively. However, for the finite value of the $\bar{Z}$, the surfaces becomes totally reacting or sound absorbent.

The Helmholtz equation


$$\left(\bar{\nabla^{2}}+k^{2}\right)\bar{\phi }=0.$$ (3)

describes the acoustic propagation in the compressible fluid, and in term of harmonic fluid potential $\bar{\phi }$. The incident radiation from extended inlet propagates towards positive *z*-direction and it will attenuate on interaction with the chamber components having membrane at the interfaces. The mathematical formulation that describes the wave propagation phenomenon is made dimensionless with respect to the length and time scale *k*^−1^ and *w*^−1^, respectively, such that $k\bar{z}=z$, $k\bar{r}=r$, $\omega \bar{t}=t$ and $k^{2}\bar{\phi }=\omega \phi$. In dimensionless setting, the fluid potential ϕ in duct regions at 0 ≤ *r* ≤ *a* can be expressed as


$$\phi \left(r,z\right)=\left\{ \begin{array}{cc} \phi_{1}\left(r,z\right), & z<-L,\\ \phi_{2}\left(r,z\right), & |z|<L,\\ \phi_{3}\left(r,z\right), & z>L. \end{array}\right.$$ (4)

Note that the acoustic and membrane fields are strongly coupled at the interfaces *z* = ±*L*. We present them in separate subsections only to keep the governing equations and notation clear. The coupling is enforced through interface conditions: the acoustic pressure jump drives the membranes, and the membrane motion provides the kinematic condition for the acoustic field. These coupling relations are summarized in Subsection 2.3.

### 2.1 Acoustic response in the duct regions

In this subsection we derive the acoustic modal expansions in each region. The acoustic waves in the duct regions can be described by formulating the associated boundary value problem. The governing equations for the acoustic response involve Helmholtz equation together with rigid and impedance conditions. The dimensionless forms of these equations are given below.

- Helmholtz equation
  $$\left(\frac{\partial^{2}}{\partial r^{2}}+\frac{1}{r}\frac{\partial }{\partial r}+\frac{\partial^{2}}{\partial z^{2}}+1\right)\phi_{j}(r,z)=0,\;j=1,2,3.$$ (5)
- Rigid conditions
  $$\frac{\partial \phi_{1}}{\partial r}=0=\frac{\partial \phi_{3}}{\partial r},\;r=a,\;|z|>L.$$ (6)
- Impedance conditions
  $$\phi_{2}-i\eta \frac{\partial \phi_{2}}{\partial r}=0\;\text{at}\;r=a\text{ and }\;|z|<L,$$ (7)

where $\eta =\bar{Z}\rho c$ is the dimensionless from of normalized impedance condition so that ℜ(η)>0 represents dissipative (resistive) lining behavior, whereas purely imaginary η corresponds to lossless reactive behavior. In the parametric study we use representative test values (e.g., η=i and η=1±i) to contrast reactive versus dissipative liner responses, while η→∞ and η=0 recover rigid and soft limits.

The eigenfunction expansion in duct regions can be expressed as


$$\phi_{1}(r,z)=e^{i(z+L)}+\sum_{n=0}^{\infty }A_{n}J_{0}(\gamma_{n}r)e^{-i\kappa_{n}\left(z+L\right)},$$ (8)

$$\phi_{2}(r,z)=\sum_{n=0}^{\infty }B_{n}J_{0}(\lambda_{n}r)e^{i\nu_{n}\left(z\right)}+\sum_{n=0}^{\infty }C_{n}J_{0}(\lambda_{n}r)e^{-i\nu_{n}\left(z\right)},$$ (9)

$$\phi_{3}(r,z)=\sum_{n=0}^{\infty }D_{n}J_{0}(\gamma_{n}r)e^{i\kappa_{n}\left(z-L\right)},$$ (10)

where $\kappa_{n}=\sqrt{1-\gamma_{n}^{2}}$ and $\nu_{n}=\sqrt{1-\lambda_{n}^{2}}$ express the nth mode wavenumbers with eigenvalues $\gamma_{n}$ and $\lambda_{n}$ to be the roots of characteristics equations


$$\gamma J_{1}(\gamma a)=0\;\text{and}\;J_{0}(\lambda a)+i\eta \lambda J_{1}(\lambda a)=0.$$

In Regions I and III the cross-section is bounded and includes the axis *r* = 0, so regularity excludes Bessel function of second kind *Y*_0_ and the guided-duct radial modes are Bessel function of first kind $J_{0}(\gamma_{n}r)$. The radiation/decay condition is imposed *axially* via the signs of the factors $e^{\pm i\kappa_{n}z}$ and exponential decay for cut-off modes. The eigenfunctions $J_{0}(\gamma_{n}r)$; for n=0,1,2,⋯ and $J_{0}(\lambda_{n}r);\;n=0,1,2,\cdots$, are orthogonal and satisfy the orthogonality relations


$$\int_{0}^{a}J_{0}(\gamma_{n}r)J_{0}(\gamma_{m}r)rdr=\delta_{mn}\Theta_{n}\;\text{and}\;\int_{0}^{a}J_{0}(\lambda_{n}r)J_{0}(\lambda_{m}r)rdr=\delta_{mn}\Upsilon_{n},$$ (11)

where


$$\Theta_{n}=\int_{0}^{a}J_{0}^{2}(\gamma_{n}r)rdr\;\text{and}\;\Upsilon_{n}=\int_{0}^{a}J_{0}^{2}(\lambda_{n}r)rdr.$$

In (8), the first term corresponds to the incident field, while the second term represents the reflected field. The coefficients {*A*_*n*_, *B*_*n*_, *C*_*n*_, *D*_*n*_} are unknown and will be determined later using the mode-matching method. Specifically, these amplitudes are fixed by applying the kinematic continuity and membrane-loading relations at *z* = ±*L* (see Section 3), together with orthogonality.

### 2.2 Structure response: Galerkin formulation

To model the vibrational response of membranes on the interfaces *z* = ±*L*, the Galerkin formulation is adopted. The dimensionless form of equations that express the dynamical response of membranes at the interfaces are


$$\left(\frac{\partial^{2}}{\partial r^{2}}+\frac{1}{r}\frac{\partial }{\partial r}+\mu^{2}\right)q_{1}=\alpha (\phi_{2}-\phi_{1}),$$ (12)

$$\left(\frac{\partial^{2}}{\partial r^{2}}+\frac{1}{r}\frac{\partial }{\partial r}+\mu^{2}\right)q_{2}=\alpha (\phi_{3}-\phi_{2}),$$ (13)

where *q*_1_ and *q*_2_ are the membrane displacements lying at interfaces *z* = ±*L*. In these equations, $\mu =c/c_{m}$ represents the membrane wavenumber, where *c*_*m*_ is the speed of wave on membrane whose value with and without perforation is given by $c_{m}=\sqrt{T/\rho_{m}}$. Note that in the present study we restrict attention to the axisymmetric setting (*m* = 0), consistent with the axisymmetric device geometry and the modal basis used in the duct expansions. Extension to azimuthal orders *m* ≠ 0 follows the same steps by employing $J_{m}(\cdot )$ radial eigenfunctions and circumferential dependence $e^{im\theta }$, but is beyond the scope of this manuscript. The quantity α is the fluid-loading parameter. For the system with membrane loading along with and without perforation $\alpha =c^{2}\rho_{0}/(kT)$. Furthermore, the membrane is connected to the rim with fixed edge conditions. These conditions yield


$$q_{j}=0,\;z=\pm L,\;r=a,\;j=1,2.$$ (14)

Note that for the derivation of (12)-(14) while starting with (1) see S1 Appendix A. To find the membrane displacements by using the Galerkin approach, we solve the eigenvalue problems associated with (12) and (13) subject to fixed edge conditions (14), to get


$$q_{1}=\sum_{n=0}^{\infty }H_{1n}\psi_{n},$$ (15)

$$q_{2}=\sum_{n=0}^{\infty }H_{2n}\psi_{n},$$ (16)

where, *H*_1*n*_ and *H*_2*n*_ are the Fourier coefficients. Here $\psi_{n}=J_{0}(\xi_{n}r)$ are eigenfunctions. The corresponding eigenvalues are the roots of dispersion relation $J_{0}(\xi_{n}a)=0$. These eigenfunctions are orthogonal and satisfy the orthogonality relation


$$\int_{0}^{a}\psi_{n}\psi_{m}rdr=F_{m}\delta_{mn}\;\text{with}\;F_{n}=\int_{0}^{a}\psi_{n}^{2}rdr\;\text{and}\;\delta_{mn}:=\left\{ \begin{array}{cc} 1 & m=n\\ 0 & m\neq n \end{array}\right..$$ (17)

The Fourier coefficients *H*_1*n*_ and *H*_2*n*_ in equations (15) and (16) are unknowns and would be found by following the mode-matching procedure explained in the next section. Although the membranes are located at different interfaces (*z* = −*L* and *z*=+*L*), they are not independent: their interaction is mediated by the acoustic field inside the central chamber. In the mode-matching formulation this appears through the shared chamber modal coefficients, so the interface conditions at *z* = ±*L* produce a coupled linear system in which the response at one membrane influences the pressure/velocity field driving the other. Therefore, Mode matching provides a complete guided-wave representation of the acoustic field in each duct segment. In the coupled problem, the membrane displacement is an unknown interfacial field; a Galerkin projection is used to express it in a finite basis and to convert the membrane equation and coupling conditions into algebraic relations. For purely acoustic junctions (no membranes), mode matching can be used on its own, for instance see [12].

## 3 Mode-matching formulation

The coefficients {*A*_*n*_, *B*_*n*_, *C*_*n*_, *D*_*n*_} involved with expansions (8)-(10) are still unknowns. To that end, we match the propagating velocity and dynamical modes at the interfaces *z* = ±*L*. By utilizing the symmetry in the configuration at *z* = 0, we may break down the problem into a symmetric problem and an anti-symmetric problem. These problems provide a convenient way of imposing matching conditions at the interfaces. In symmetric problem we consider a rigid disc at *z* = 0 while in the anti-symmetric case we consider a soft disc at the interface *z* = 0. The mode-matching formulation in both of these problems is discussed in the following sub-sections.

### 3.1 Symmetric problem

The consideration of the rigid disc at *z* = 0 yields *B*_*n*_ = *C*_*n*_. We use superscript “*s*” with the variables while considering the symmetric setting. The eigenfunction expansions (8)-(10) for the symmetric setting can be expressed as


$$\phi_{1}^{s}(r,z)=e^{i(z+L)}+\sum_{n=0}^{\infty }A_{n}^{s}J_{0}(\gamma_{n}r)e^{-i\kappa_{n}\left(z+L\right)},$$ (18)

$$\phi_{2}^{s}(r,z)=\sum_{n=0}^{\infty }2B_{n}^{s}\cos(\nu_{n}z)J_{0}(\lambda_{n}r).$$ (19)

Further, the formulation to determine the behavior of membranes at interfaces in the symmetric setting can be given as


$$q^{s}(r)=\sum_{n=0}^{\infty }H_{n}^{s}J_{0}(\xi_{n}r),$$ (20)

where $H_{n}^{s}$ expresses the symmetric mode amplitude of membrane disc and is to be determined. To that end, we utilize the symmetric form of the membrane disc at *z* = −*L*, that is


$$\left(\frac{\partial^{2}}{\partial r^{2}}+\frac{1}{r}\frac{\partial }{\partial r}+\mu^{2}\right)q^{s}(r)=\alpha (\phi_{2}^{s}-\phi_{1}^{s}).$$ (21)

On using formulation (20) along with (18) and (19), we get


$$\sum_{n=0}^{\infty }H_{n}^{s}\Delta_{n}J_{0}(\xi_{n}r)=\sum_{n=0}^{\infty }2\alpha B_{n}^{s}\cos(\nu_{n}L)J_{0}(\lambda_{n}r)-\alpha -\alpha \sum_{n=0}^{\infty }A_{n}^{s}J_{0}(\gamma_{n}r).$$ (22)

On multiplying with $J_{0}(\xi_{m}r)r$, integrating with respect to *r* from 0 to *a*, we find


$$H_{m}^{s}=\frac{\alpha }{F_{m}\Delta_{m}}\left\{-\Lambda_{1m0}-\sum_{n=0}^{\infty }A_{n}^{s}\Lambda_{1mn}+\sum_{n=0}^{\infty }2B_{n}^{s}\cos(\nu_{n}L)\Lambda_{2mn}\right\},$$ (23)

where


$$\Lambda_{1mn}=\int_{0}^{a}J_{0}(\gamma_{n}r)J_{0}(\xi_{m}r)rdr\;\text{and}\;\Lambda_{2mn}=\int_{0}^{a}J_{0}(\lambda_{n}r)J_{0}(\xi_{m}r)rdr,$$

with $\Delta_{m}=\mu^{2}-\xi_{m}^{2}$. Now, we use the continuity of the normal velocities at the membrane interface *z* = −*L*, i.e.,


$$\frac{\partial \phi_{1}^{s}}{\partial z}(r,-L)=\frac{\partial \phi_{2}^{s}}{\partial z}(r,-L)=q^{s}(r),\;0<r<a.$$ (24)

Considering $\phi_{1z}^{s}(r,-L)=q^{s}(r)$, using (18) and (20) above, multiplying the result with $J_{0}(\gamma_{m}r)r$, integrating with respect to *r* from 0 to *a*, and using (11), we obtain


$$A_{m}^{s}=\delta_{m0}-\frac{1}{i\kappa_{m}\Theta_{m}}\sum_{n=0}^{\infty }H_{n}^{s}\Lambda_{1nm},$$ (25)

Likewise, by considering $\phi_{2z}^{s}(r,-L)=q^{s}(r)$, substituting (19) and (20), multiplying with $J_{0}(\lambda_{m}r)r$, integrating with respect to *r* from 0 to *a*, and using (11), we arrive at


$$B_{m}^{s}=-\frac{1}{2\sin(\nu_{m}L)\nu_{m}\Upsilon_{m}}\sum_{n=0}^{\infty }H_{n}^{s}\Lambda_{2nm}.$$ (26)

On using (25) and (26) into (23), we get the following linear algebraic system


$$H_{m}^{s}=\frac{\alpha }{F_{m}{△}_{m}}\left\{-2\Lambda_{1m0}-i\sum_{n=0}^{\infty }\sum_{p=0}^{\infty }\frac{H_{p}^{s}\Lambda_{1pn}\Lambda_{1mn}}{\kappa_{n}\Theta_{n}}-\sum_{n=0}^{\infty }\sum_{p=0}^{\infty }\frac{H_{p}^{s}\cos(\nu_{n}L)\Lambda_{2pn}\Lambda_{2mn}}{\sin(\nu_{n}L)\nu_{n}\Upsilon_{n}}\right\}.$$ (27)

For m=n=p=0,1,2,⋯, the system defined by (27) leads to a consistent system which is truncated and solved numerically.

### 3.2 Anti-symmetric problem

For the anti-symmetric setting, we may assume a soft disc located at *z* = 0 which yields *C*_*n*_ = −*B*_*n*_. Further, we may differentiate the variables in this case with superscript “*a*.” The eigenfunction expansions for the the anti-symmetric configuration can be given as


$$\phi_{1}^{a}(r,z)=e^{i(z+L)}+\sum_{n=0}^{\infty }A_{n}^{a}J_{0}(\gamma_{n}r)e^{-i\kappa_{n}\left(z+L\right)},$$ (28)

$$\phi_{2}^{a}(r,z)=\sum_{n=0}^{\infty }B_{n}^{a}2i\sin(\nu_{n}z)J_{0}(\lambda_{n}r).$$ (29)

We assume the displacement with the undetermined Fourier coefficients $H_{n}^{a}$,


$$q^{a}(r)=\sum_{n=0}^{\infty }H_{n}^{a}J_{0}(\xi_{n}r).$$ (30)

The membrane displacement *q*^*a*^(*r*) satisfies the equation of motion,


$$\left(\frac{\partial^{2}}{\partial r^{2}}+\frac{1}{r}\frac{\partial }{\partial r}+\mu^{2}\right)q^{a}(r)=\alpha (\phi_{2}^{a}-\phi_{1}^{a}),$$ (31)

which on using (28) and (29) along with (30), and on some mathematical simplifications leads to


$$H_{m}^{a}=\frac{\alpha }{F_{m}\Delta_{m}}\left\{-\Lambda_{1m0}-\sum_{n=0}^{\infty }A_{n}^{a}\Lambda_{1mn}-\sum_{n=0}^{\infty }2iB_{n}^{a}\sin(\nu_{n}L)\Lambda_{2mn}\right\}.$$ (32)

To determine the anti-symmetric mode amplitudes $\left\{A_{n}^{a},B_{n}^{a}\right\}$, we consider the matching conditions,


$$\frac{\partial \phi_{1}^{a}}{\partial z}(r,-L)=\frac{\partial \phi_{2}^{a}}{\partial z}(r,-L)=q^{a}(r),\;0<r<a.$$ (33)

Adopting the procedure as explained in the symmetric problem, this yields the expression


$$A_{m}^{a}=\frac{\delta_{m0}}{\kappa_{m}}-\frac{1}{i\kappa_{m}\Theta_{m}}\sum_{n=0}^{\infty }H_{n}^{a}\Gamma_{2mn},$$ (34)

$$B_{m}^{a}=\frac{1}{2i\cos(\nu_{m}L)\nu_{m}\Upsilon_{m}}\sum_{n=1}^{\infty }H_{n}^{a}\Gamma_{1mn},$$ (35)

for the anti-symmetric mode amplitudes. By using (34) and (35) into (32), we get the following linear algebraic system


$$H_{m}^{a}=\frac{\alpha }{F_{m}{△}_{m}}\left\{-2\Lambda_{1m0}-i\sum_{n=0}^{\infty }\sum_{p=0}^{\infty }\frac{H_{p}^{a}\Lambda_{1pn}\Lambda_{1mn}}{\kappa_{n}\Theta_{n}}+\sum_{n=0}^{\infty }\sum_{p=0}^{\infty }\frac{H_{p}^{a}\sin(\nu_{n}L)\Lambda_{2pn}\Lambda_{2mn}}{\cos(\nu_{n}L)\nu_{n}\Upsilon_{n}}\right\}.$$ (36)

For m=n=p=0,1,2,⋯, (36) leads to a consistent system with unknowns $H_{m}^{a}$. This system is truncated and solved numerically. Once $H_{m}^{a}$, for m=0,1,2,⋯, are known, (34) and (35) will yield the anti-symmetric modes.

## 4 Scheme validation and convergence

Here, the proposed numerical scheme is validated and the convergence of the scheme is discussed in terms of the power and transmission loss. The main trends in the numerical results can be understood through (i) cut-on of higher-order duct modes, (ii) interaction between localized chamber resonances and propagating duct modes, (iii) frequency-dependent compliance of the membrane interfaces, and (iv) impedance matching versus dissipation in the lined section.

### 4.1 Validation of the scheme

The infinite systems of equations derived for the symmetric and anti-symmetric sub-problems are first truncated to *N* terms and then solved numerically. The truncated systems are made consistent by setting *m* = *n*, resulting in *N* + 1 equations with an equal number of unknown amplitudes. The computations are carried out in *Mathematica*: the eigenvalues are obtained by built-in root-finding (e.g., NSolve/FindRoot), the coupling integrals are evaluated numerically (e.g., NIntegrate), and the resulting dense linear systems are solved using built-in linear-algebra solvers (e.g., LinearSolve). To perform the numerical computations, the speed of sound in air is taken as *c* = 344 ms^−1^. The truncated amplitudes {*H*_*n*_, *A*_*n*_, *B*_*n*_, *C*_*n*_, *D*_*n*_}, which appear in the expansions (8)-(10), can be obtained from the unknowns of the symmetric systems $\left\{H_{n}^{s},A_{n}^{s},B_{n}^{s},C_{n}^{s},D_{n}^{s}\right\}$ and the anti-symmetric systems $\left\{H_{n}^{a},A_{n}^{a},B_{n}^{a},C_{n}^{a},D_{n}^{a}\right\}$ as


$$H_{1n}=\frac{H_{n}^{s}+H_{n}^{a}}{2},\;H_{2n}=\frac{H_{n}^{s}-H_{n}^{a}}{2},$$

$$A_{n}=\frac{A_{n}^{s}+A_{n}^{a}}{2},\;D_{n}=\frac{A_{n}^{s}-A_{n}^{a}}{2},$$

$$B_{n}=\frac{B_{n}^{s}+B_{n}^{a}}{2},\;C_{n}=\frac{B_{n}^{s}-B_{n}^{a}}{2}.$$

By determining the truncated amplitudes, (8)-(10) and (15)-(16) yield the fluid potentials and membrane displacements, respectively. The accuracy of these truncated formulations can be assessed by reconstructing the matching conditions that were initially imposed to derive the linear algebraic systems. For this purpose, we truncate the systems by considering *N* = 51 terms and fix the configuration with a radius of $\bar{a}=0.2$ m and a chamber half-length of $\bar{L}=0.2$ m.

**Fig 2:**
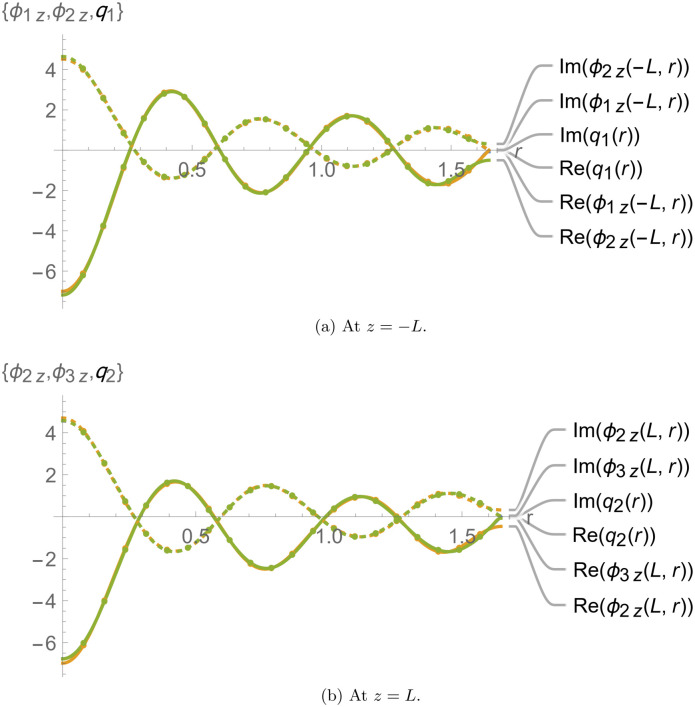
Membrane displacements and normal velocities at *z* = ±*L.*

**Fig 3:**
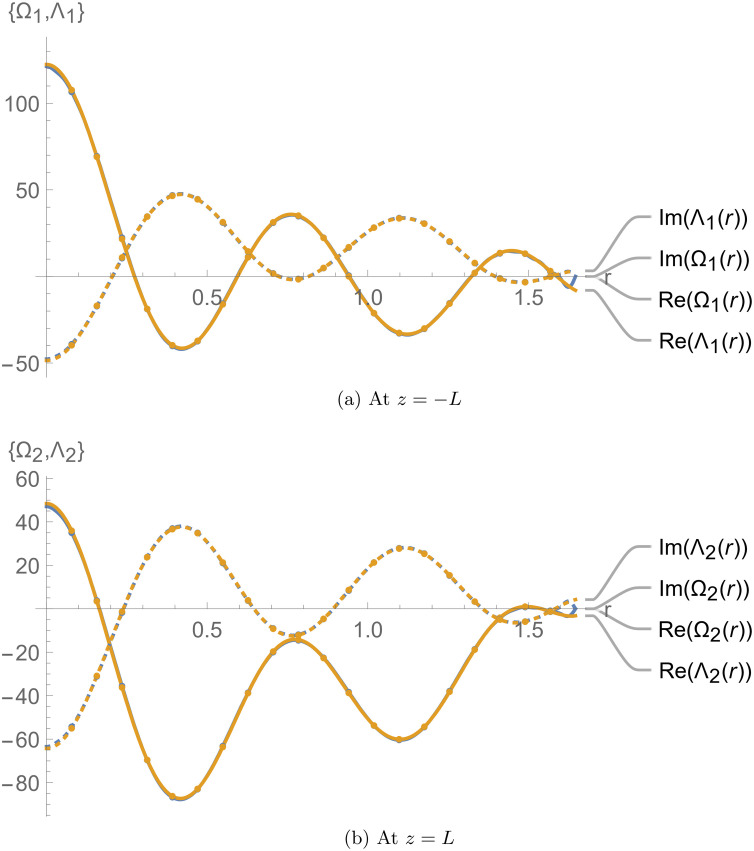
Elastic membrane conditions at *z* = ±*L.*

Figs 2 and 3 depict the continuity conditions at the interfaces *z* = ±*L*. We specifically applied the matching conditions $\phi_{1z}(r,z)=\phi_{2z}(r,z)=q_{1}$ and $\phi_{2z}(r,z)=\phi_{3z}(r,z)=q_{2}$ for 0 ≤ *r* ≤ *a* to determine the unknown amplitudes, leading to the truncated solution. The numerical reconstruction of these conditions based on the truncated solution is shown in Fig 2, with subfigures 2a and 2b displaying the results at *z* = −*L* and *z* = *L*, respectively. The curves align perfectly at the interfaces, confirming that the truncated solution satisfies the imposed matching conditions.

Additionally, to verify if the truncated solution satisfies the membrane equation, we rewrite equations (12) and (13) as follows:


$$\Lambda_{1}(r):=\alpha (\phi_{2}-\phi_{1}),\;\Lambda_{2}(r)=\alpha (\phi_{3}-\phi_{2}),$$ (37)

$$\Omega_{1}(r):=w_{1rr}+\mu^{2}w_{1},\;\;\;\Omega_{2}(r)=w_{2rr}+\mu^{2}w_{2}.$$ (38)

In Fig 3, $\Lambda_{j}(r)$ and $\Omega_{j}(r)$ are plotted against *r*, and the alignment of the curves confirms that the truncated solution satisfies the matching conditions defined in (12) and (13).

### 4.2 Convergence of the scheme

The power or energy flux can be found from the field by using the definition (see, for instance, [27])


$$\text{Energy flux}=\frac{1}{2}\text{Re}\left[\int_{0}^{a}i\phi {\left(\frac{\partial \phi }{\partial n}\right)}^{*}d\Sigma \right],$$ (39)

By considering the incident, reflected, and transmitted fields, the power identity is obtained by applying the conservation law of energy and normalizing the incident power to unity as


$${ℰ}_{1}+{ℰ}_{2}=1,$$ (40)

where


$${ℰ}_{1}=ℜ\left\{\sum_{m=0}^{𝒦-1}|A_{m}{|}^{2}\Theta_{m}\kappa_{m}\right\},$$ (41)

$${ℰ}_{2}=ℜ\left\{\sum_{m=0}^{𝒦-1}|D_{m}{|}^{2}\Theta_{m}\kappa_{m}\right\}.$$ (42)

Here, ${ℰ}_{1}$ and ${ℰ}_{2}$ express the reflected and transmitted powers, respectively, whereas 𝒦 expresses the number of cut-on modes. However, the expression for the absorbed energy is ${ℰ}_{\text{abs}}=1-{ℰ}_{1}-{ℰ}_{2}$. The transmission loss can be defined as (see, for instance, [26])


$$\text{Transmission Loss (TL)}=-10\log_{10}({ℰ}_{2}).$$ (43)

**Fig 4:**
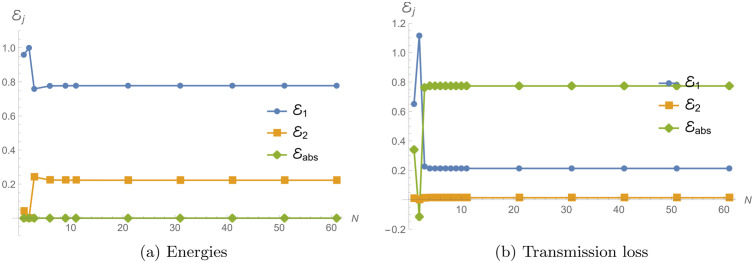
Convergence of power components and transmission loss vs. truncation number *N.*

The convergence of reflecting, transmitting and absorbed energies versus number of terms is substantiated in Table 1 and Fig 4. The convergence study of energy components and TL is conducted for two different boundary conditions: an impedance condition (η=i) and an absorbent condition (η=1+i). The analysis focuses on the behavior of these quantities as the truncation number *N* varies, with parameters $\bar{a}=0.2\;\text{m}$, $\bar{L}=0.2\;\text{m}$, and frequency *f* = 300 Hz. Note that all plots are shown in normalized form (relative to the incident-field amplitude) to emphasize modal patterns; axes are dimensionless unless stated otherwise. Accordingly, we use *N* = 31 in all computations: for example, in Table 1 increasing from *N* = 31 to *N* = 61 changes the energy components only in the sixth decimal place and changes the transmission loss by less than 2 × 10^−4^ dB for the representative cases considered.

**Table 1:** Convergence of energies when $\bar{a}=0.2$m, $\bar{L}=0.2$m, and *f* = 300 Hz.

Cases	*N*	${ℰ}_{1}$	${ℰ}_{2}$	${ℰ}_{1}+{ℰ}_{2}$	${ℰ}_{abs}$	TL
	1	0.958776	0.0412245	1	0	13.8484
Impedance condition	2	0.999399	0.00060138	1	0	32.2085
(η=i)	3	0.758441	0.241559	1	0	6.16977
	6	0.776229	0.223771	1	0	6.50197
	11	0.777274	0.222726	1	0	6.52230
	31	0.777458	0.222542	1	0	6.52587
	51	0.777464	0.222536	1	0	6.52610
	61	0.77746	0.222535	1	0	6.52601
	1	0.650916	0.00895259	0.659868	0.340132	20.4805
Absorbent condition	2	1.11502	0.000262249	1.11528	−0.115278	35.8129
(η=1+i)	3	0.226145	0.0118039	0.237948	0.762052	19.2798
	6	0.212993	0.0137145	0.226707	0.773293	18.6282
	11	0.212966	0.0137779	0.226744	0.773256	18.6082
	31	0.212956	0.0137881	0.226744	0.773256	18.6050
	51	0.212956	0.0137884	0.226744	0.773256	18.6049
	61	0.212956	0.0137884	0.226744	0.773256	18.6049

Under the impedance condition (η=i), the energy components ${ℰ}_{1}$ and ${ℰ}_{2}$ exhibit rapid convergence as *N* increases. Initially, ${ℰ}_{1}=0.958776$ at *N* = 1, and it converges to approximately 0.777460 at *N* = 61. The second energy component, ${ℰ}_{2}$, starts with a small value of 0.0412245 at *N* = 1 and converges to about 0.222535 at *N* = 61. The total energy, ${ℰ}_{1}+{ℰ}_{2}$, remains consistently equal to 1, which indicates that energy conservation is maintained within the system. Notably, there is no absorbed energy, ${ℰ}_{\text{abs}}=0$, under the impedance condition, which is expected since this condition does not dissipate energy. TL under the impedance condition starts at a relatively high value of 13.8484 dB for *N* = 1 and increases significantly to 32.2085 dB at *N* = 2. As *N* increases further, the TL decreases and converges around 6.52601 dB at *N* = 61.

In contrast, for the absorbent condition (η=1+i), the energy components converge differently. Initially, ${ℰ}_{1}=0.650916$ and ${ℰ}_{2}=0.00895259$ at *N* = 1. Both components gradually converge to approximately ${ℰ}_{1}=0.212956$ and ${ℰ}_{2}=0.0137884$ as *N* reaches 61. Additionally, the absorbed energy, ${ℰ}_{\text{abs}}$, increases as *N* increases, starting from 0.340132 at *N* = 1 and converging to around 0.773256 at *N* = 61. TL starting at 20.4805 dB for *N* = 1, increases to 35.8129 dB at *N* = 2, then gradually decreases and converges to around 18.6049 dB at *N* = 61.

In both cases, the energy components and TL exhibit convergence as the truncation number *N* increases. For both conditions, the energy components stabilize around *N* = 31, indicating that further increases in *N* yield diminishing changes in the computed values. The impedance condition shows a faster convergence of TL compared to the absorbent condition, though both conditions eventually reach stable values. Overall, the truncated solution is convergent when *N* > 10, see, for example, Fig 4, or Table 1. Further, we note that for very small truncation levels, the truncated system can temporarily produce non-physical power splits (e.g., negative inferred absorbed energy or totals exceeding unity). These are truncation artifacts that disappear as the modal order is increased; for this reason, all subsequent computations use a truncation level for which the power components and TL are converged.

## 5 Numerical results and discussion

We perform some numerical experiments to elaborate the influence of different parameters and functions on the power transmission and reflection of the waveguide structure.

### 5.1 Power and transmission loss vs. frequency

**Fig 5:**
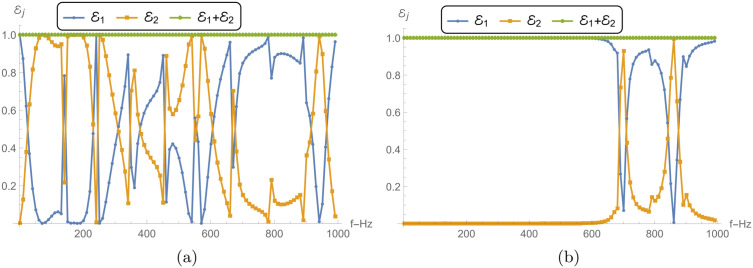
Reflected power ${ℰ}_{1}$, transmitted power ${ℰ}_{2}$ and their sum ${ℰ}_{1}+{ℰ}_{2}$ against frequency with different chamber conditions: (a) Rigid conditions and (b) Soft conditions, where $\bar{a}=0.2$m, $\bar{L}=0.2$m, and *N* = 31 terms.

**Fig 6:**
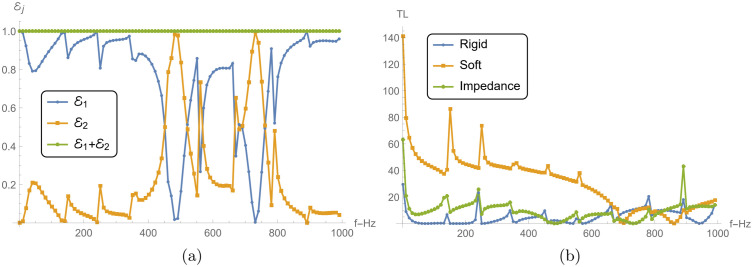
Power components and TL aginst frequency. (a) Reflected power ${ℰ}_{1}$, transmitted power ${ℰ}_{2}$ and their sum ${ℰ}_{1}+{ℰ}_{2}$ against frequency with different impedance chamber condition. (b) TL against frequency with rigid, soft and impedance chamber condition.

Figs 5 and 6 present the reflected power ${ℰ}_{1}$, transmitted power ${ℰ}_{2}$, their sum ${ℰ}_{1}+{ℰ}_{2}$, and the TL across different chamber conditions, including rigid, soft, and impedance boundaries. The analysis is conducted for frequencies ranging from 0 to 1000 Hz, with parameters $\bar{a}=0.2\;\text{m}$, $\bar{L}=0.2\;\text{m}$, and *N* = 31 terms.

Fig 5a illustrates the reflected and transmitted power as functions of frequency for a rigid boundary condition. The reflected power ${ℰ}_{1}$ exhibits strong oscillatory behavior across the frequency range, periodically reaching values close to 1. This indicates that most of the incident energy is being reflected at certain frequencies, leading to minimal transmission. The transmitted power ${ℰ}_{2}$, in contrast, shows complementary oscillations, peaking where ${ℰ}_{1}$ dips, suggesting that transmission primarily occurs at frequencies where reflections are minimized. These notches/dips occur near cut-on of higher-order modes and membrane–chamber resonant frequencies. At these frequencies the modal contributions in the transmitted field can interfere destructively, so ${ℰ}_{2}$ drops while ${ℰ}_{1}$ increases (complementary behavior). The sum of the reflected and transmitted power ${ℰ}_{1}+{ℰ}_{2}$ remains consistently equal to 1 throughout the frequency range, which indicates energy conservation within the system. This consistency further confirms that no energy is being absorbed or dissipated, as expected for a rigid boundary condition.

Fig 5b shows the results for a soft boundary condition. The reflected power ${ℰ}_{1}$ remains close to 1 over most of the frequency range, except for a narrow band at higher frequencies where it decreases sharply. This suggests that most of the energy is being reflected from the membrane disc as confirmed by the low values of transmitted power ${ℰ}_{2}$. The transmitted power remains nearly 0 for a wide range of frequencies, indicating minimal transmission and maximum reflection, which is a characteristic of a soft boundary in the low frequency regime. Again, the sum ${ℰ}_{1}+{ℰ}_{2}$ remains at 1 across all frequencies, reaffirming the conservation of energy, with no absorption in the system under this boundary condition.

Fig 6a shows the reflected and transmitted power for the impedance boundary condition. Similar to the rigid condition, ${ℰ}_{1}$ and ${ℰ}_{2}$ exhibit oscillatory behavior, but with different amplitudes and frequency characteristics. The reflected power ${ℰ}_{1}$ does not reach the same high peaks as in the soft case, indicating that the impedance boundary allows more energy to be transmitted. However, there are still frequencies where transmission dominates, albeit to a lesser extent in low frequency regime. The transmitted power ${ℰ}_{2}$ complements the behavior of ${ℰ}_{1}$, with the sum ${ℰ}_{1}+{ℰ}_{2}$ consistently equal to 1, ensuring energy conservation. The impedance boundary strikes a balance between reflection and transmission, unlike the rigid and soft boundaries, which tend to either fully reflect or fully transmit energy, respectively.

Fig 6b compares the TL across the three boundary conditions: rigid, soft, and impedance. The rigid boundary condition exhibits very low TL across the entire frequency range. The TL remains close to zero, indicating that the majority of the incident energy is transmitted through the boundary, with minimal reflection, as seen in Fig 5a. The soft boundary, on the other hand, shows multiple sharp peaks in TL, particularly at low frequencies, where the TL reaches values above 120 dB. This indicates that very little energy is transmitted, corresponding to the strong reflections observed in Fig 5b. As the frequency increases, the TL decreases, though it still remains significant due to the oscillatory nature of the reflected power. The impedance boundary condition shows intermediate behavior, with TL values higher than those of the rigid boundary but lower than those of the soft boundary. The TL curve for the impedance condition also displays oscillations, though the peaks are lower in magnitude compared to the soft condition. This reflects the balance between reflection and transmission that characterizes the impedance boundary.

### 5.2 Potentials vs. excitation modes

**Fig 7:**
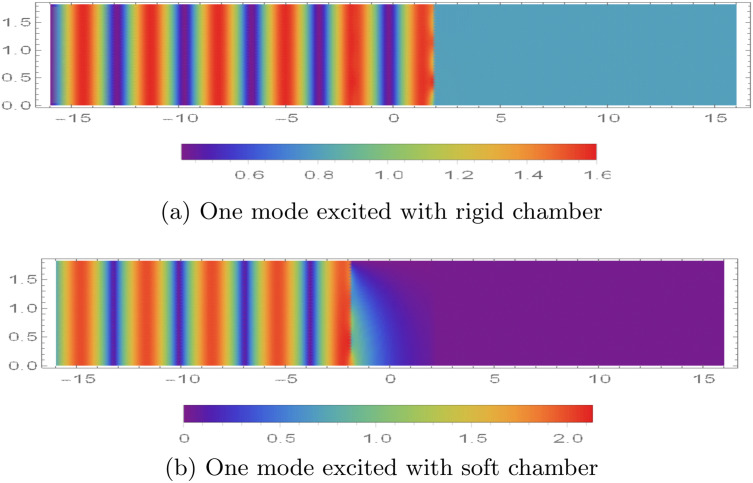
Absolute value of fluid potential |ϕ(r,z)|, for 0 < *r* < 0.2m, |*z*| < 16 when the duct is excited by single mode from z<−L.

In Fig 7, we plot the magnitude of the dimensionless acoustic potential, |ϕ(r,z)|, over 0 < *r* < *a* and |z|<16 for different *incident (excitation) duct modes* launched from the upstream side (*z* < −*L*). Here, “excitation mode” means that one incoming guided mode in Region I is prescribed (unit amplitude), while the other incoming modal amplitudes are set to zero; the reflected and transmitted modal contents are then generated by scattering at the chamber and membrane interfaces. We set $\bar{a}=0.2\;m$, $\bar{L}=0.2\;m$, and truncate the modal expansions at *N* = 31.

The contour patterns in |ϕ| represent the spatial structure created by modal superposition. Bands (or alternating high/low regions) in *z* indicate interference between forward- and backward-travelling components, while the radial variation reflects the Bessel-type mode shapes. Near cut-on/cut-off transitions, the field can change rapidly because additional modes begin to propagate or become evanescent, which alters the interference pattern. Similar interpretations of mode-matching contour maps in lined waveguides (including how modal content and interference shape the plotted magnitude fields) can be found in [42].

**Fig 8:**
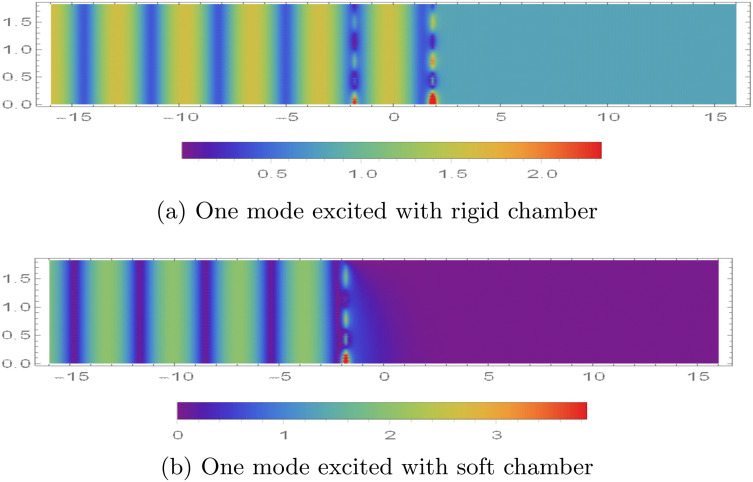
Absolute value of normal velocity $\left|\phi_{z}(r,z)\right|$, for 0 < *r* < 0.2m, |*z*| < 16 when the duct is excited by single mode from z<−L.

Figs 7a and 7b show the response for a single incident-mode excitation at *f* = 500 Hz under the two mid-plane conditions (rigid and soft). In the rigid case, the incident mode is only partially attenuated and a non-negligible field persists into the downstream region (*z* > *L*), indicating transmission through the device (Fig 7a). In the soft case, the pressure-release constraint promotes stronger suppression of the field beyond the chamber, so the transmitted magnitude in *z* > *L* is markedly reduced (Fig 7b). For completeness, the corresponding *normal (axial) velocity* patterns, based on |∂ϕ/∂z|, are shown in Fig 8, which highlights the nodal/antinodal structure associated with the same modal content.

The normal velocity modes in the waveguide are illustrated in Fig 8, using the same numerical parameter settings as those employed in Fig 7. As shown in Fig 8a, under rigid boundary conditions, distinct velocity modes appear at both interfaces. These modes are not filtered by the membrane discs and thus contribute to sound transmission, as also observed in Fig 7a. In contrast, for soft boundary conditions, velocity modes are present at the interface *z* = −*L* but are effectively filtered out at *z* = *L*, as seen in Fig 8b.

### 5.3 Power and transmission loss vs. liner conditions

**Fig 9:**
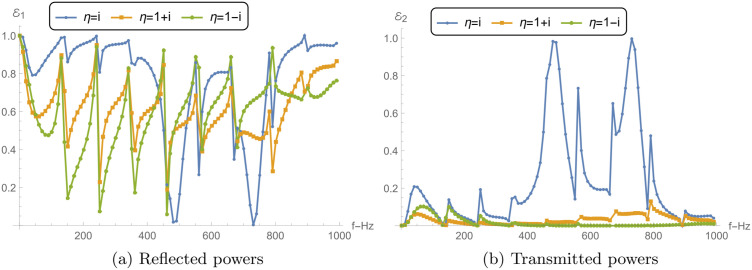
For reacting liner condition and dissipating conditions: **(a)** Reflected power $\left({ℰ}_{1}\right)$ against frequency, and **(b)** Transmitted power $\left({ℰ}_{2}\right)$ against frequency, where $\bar{a}=0.2\;\text{m}$, $\bar{L}=0.2\;\text{m}$, and *N* = 31 terms.

**Fig 10:**
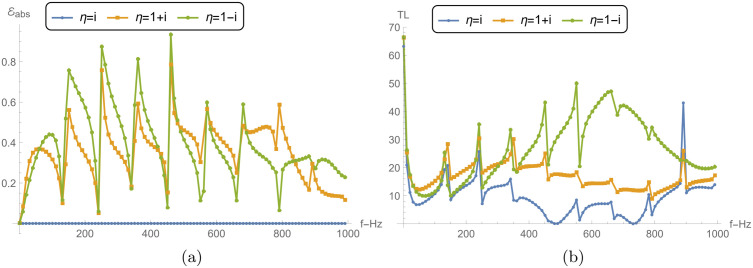
For reacting liner condition and dissipating conditions: **(a)** Absorbed power (*E*_abs_) against frequency, and **(b)** Transmission loss (TL) against frequency, where $\bar{a}=0.2\;\text{m}$, $\bar{L}=0.2\;\text{m}$, and *N* = 31 terms.

Figs 9 and 10 explore the reflected power ${ℰ}_{1}$, transmitted power ${ℰ}_{2}$, absorbed power *E*_abs_, and transmission loss (TL) for a reactive liner and two dissipative liners. The calculations correspond to $\bar{a}=0.2\;\text{m}$, $\bar{L}=0.2\;\text{m}$, and *N* = 31, over the frequency range 0 ≤ *f*≤ 1000 Hz. Recall that η=i is purely reactive (lossless), whereas η=1±i has a resistive part and therefore permits absorption (*E*_abs_ > 0). Fig 9a shows the reflected power ${ℰ}_{1}$ versus frequency. For the reactive liner, ${ℰ}_{1}$ varies noticeably with frequency and exhibits peaks approaching 1, together with distinct dips at several frequencies. Since this case is lossless, these variations mainly reflect redistribution of energy between reflection and transmission rather than dissipation. For the dissipative liners, the reflection peaks are generally reduced and the fluctuations are more damped because a portion of the incident energy is removed by absorption. Between the two dissipative cases, η=1+i can show slightly higher reflection than η=1−i. This indicates that, relative to η=1−i, less energy is diverted into absorption (and hence more remains available to be reflected), which is consistent with the absorption levels shown in Fig 10a.

Fig 9b presents the transmitted power ${ℰ}_{2}$. For the reactive liner, ${ℰ}_{2}$ exhibits sharp transmission peaks that align with the dips in ${ℰ}_{1}$ in Fig 9a, consistent with a primarily lossless exchange between reflected and transmitted components. For the dissipative liners, ${ℰ}_{2}$ is generally lower and the sharp peaks are suppressed, because part of the incident energy is dissipated in the lining, leaving less energy available for transmission.

Fig 10a shows the absorbed power *E*_abs_. As expected, the reactive liner yields *E*_abs_ = 0 across the entire band, confirming that it does not dissipate energy. In contrast, both dissipative liners exhibit significant absorption over the frequency range. The case η=1−i shows higher absorption peaks than η=1+i across much of the band, indicating stronger dissipation for η=1−i.

Fig 10b presents the transmission loss. The reactive liner produces moderate TL with oscillatory peaks, reflecting the frequency-dependent exchange between reflection and transmission in the absence of absorption. The dissipative liners yield substantially higher TL because absorption reduces the transmitted power. The case η=1−i gives the largest TL peaks (exceeding 48 dB), consistent with its higher absorbed power in Fig 10a, while η=1+i also produces high TL values but typically lower than η=1−i. Overall, the dissipative conditions reduce transmission primarily by enhancing absorption and damping the sharp frequency-dependent features observed in the reactive case. Nevertheless, the local dips/spikes in ${ℰ}_{1}$ and ${ℰ}_{2}$ are mainly driven by modal cut-on and junction/membrane resonances, which redistribute energy between reflection and transmission. When a dissipative liner is used (ℜ(η)>0), these sharp features are damped and the curves become smoother because part of the energy is removed as *E*_abs_.

**Fig 11:**
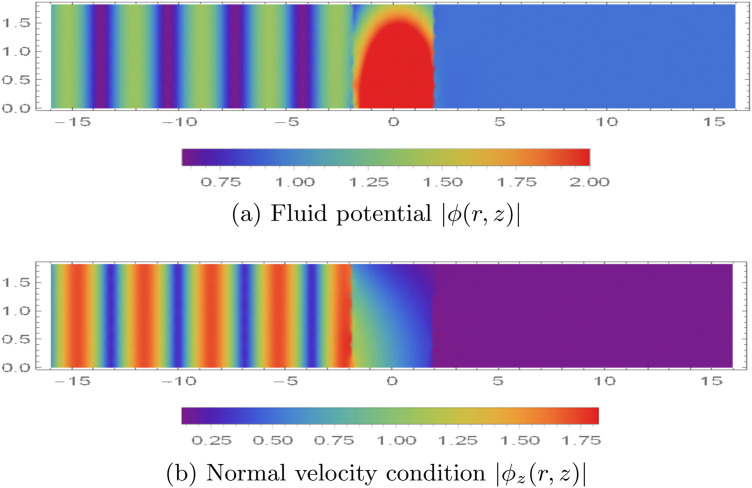
For reacting condition, the absolute value of fluid potential and normal velocity with 0 < *r* < 0.2m and |*z*| < 16.

**Fig 12:**
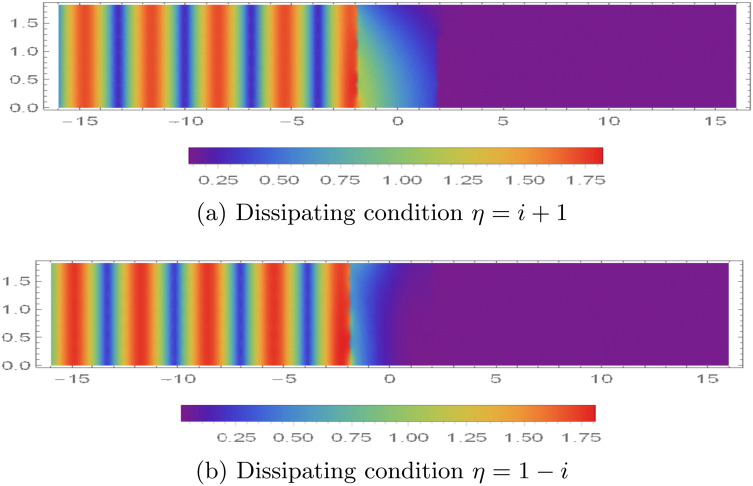
Absolute value of fluid potential |ϕ(r,z)| with 0 < *r* < 0.2m and |*z*| < 16 for dissipating conditions.

**Fig 13:**
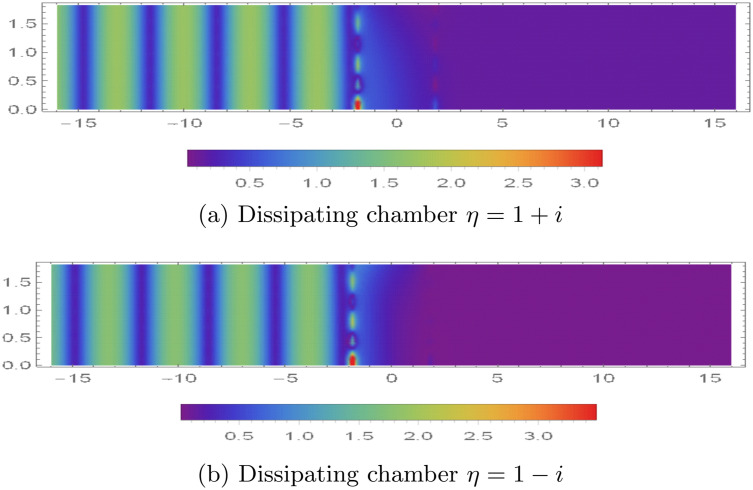
Absolute value of velocity potential $\left|\phi_{z}(r,z)\right|$ with 0 < *r* < 0.2m and |*z*| < 16 for dissipating conditions.

The spatial distribution of the absolute value of the fluid potential |ϕ(r,z)| and normal velocity $\left|\phi_{z}(r,z)\right|$ within the range 0 < *r* < 0.2 m and |*z*| < 16, are illustrated in Figs 11-13 for different acoustic lining conditions—specifically reacting and dissipative types.

In the case of reacting boundary conditions, shown in Fig 11a, certain wave energy components are able to propagate through the first membrane barrier located at *z* = −*L*. These transmitted energy modes, however, experience attenuation due to the dynamic response of a second membrane disc also positioned at *z* = −*L*. This attenuation effect is reflected in Fig 11b, where distinct velocity mode structures are observed near *z* = −*L*. The presence of these modes indicates that the membrane is actively participating in the energy transmission process by vibrating in response to the incoming fluid potential.

When the boundary condition is altered from reacting to dissipative material Figs 12 and 13 are found. By assigning a complex impedance value of η=1+i, the behavior of the acoustic field changes significantly see for instance Fig 12a. Under this dissipative condition, a considerable portion of the incident acoustic modes is absorbed at the barrier located at *z* = −*L*, resulting in a marked reduction of transmitted energy. The remaining acoustic energy that manages to pass through is eventually reflected or attenuated at the opposite barrier at *z* = *L*. As a result, Fig 13a reveals pronounced vibrational activity in the vicinity of *z* = −*L*, with only a minimal and localized motion of the membrane disc at *z* = *L*, suggesting effective damping of the acoustic field before it can fully interact with the second barrier. Further modifications to the lining properties are considered in Fig 12b, where the impedance is changed to η=1−i, introducing a different form of dissipative interaction. This configuration leads to even stronger attenuation of the acoustic energy at *z* = −*L*. The filtering effect in this case is enhanced to such an extent that virtually no energy reaches the membrane at *z* = *L*. This is clearly evidenced in Fig 13b, where the absence of visible velocity modes near *z* = *L* implies that the membrane remains essentially motionless due to the lack of incident acoustic excitation.

### 5.4 Power and transmission loss vs. waveguide radius

**Fig 14:**
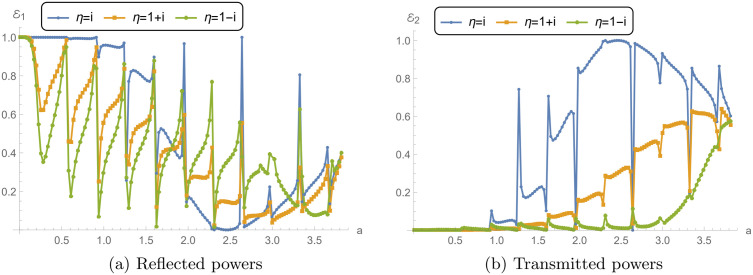
Reflected and transmitted powers vs. $a=k\bar{a}$ for reacting liner condition and dissipating conditions.

**Fig 15:**
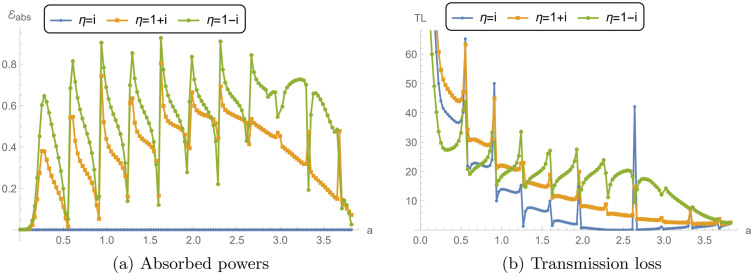
Absorbed power and transmission loss vs. $a=k\bar{a}$ for reacting liner condition and dissipating conditions.

Figs 14 and 15 present the reflected, transmitted, and absorbed power *E*_abs_, and TL as functions of the dimensionless parameter $a=k\bar{a}$. The frequency is fixed at *f* = 300 Hz and *N* = 31. The analysis focuses on reactive liner and dissipative liners.

Fig 14a shows the reflected power ${ℰ}_{1}$ as a function of *a* for the different boundary conditions. The reactive liner decreases from 1 having smooth behavior in the start and then sharp oscillations in reflected power, with several peaks reaching near 1. These peaks suggest that, for certain chamber radii, the reactive liner primarily reflects the incident wave energy back, leading to minimal energy transmission through the system. The dissipative conditions exhibit lower peaks and more damped oscillations in reflected power compared to the reactive liner. The dissipative condition η=1+i shows lesser reflection than η=1−i which is lesser than reacting liner condition. In Fig 14b, the transmitted power ${ℰ}_{2}$ is presented. For the reactive liner condition, the transmission power shows multiple sharp spikes, which correspond to the dips in the reflected power seen in Fig 14a. This implies that transmission is maximized at frequencies where the reflection is minimized, resulting in a periodic energy transfer through the chamber. For the dissipative liners, the transmitted power remains relatively lower and more stable in the start and then exhibits increasing behavior.

Fig 15a illustrates the absorbed power *E*_abs_ for the three boundary conditions. The reactive liner absorbs no energy across the entire range of *a*, confirming that the primary behavior of this boundary condition is reflection and transmission, with minimal absorption of the incident energy. In contrast, the dissipative liners show much higher absorption of energy, particularly at higher values of *a*. The condition η=1−i exhibits the highest peaks in absorbed power, suggesting that this condition is most effective at dissipating energy as the chamber radius increases. The condition η=1+i also shows considerable absorption, although slightly lower than η=1−i, indicating that the energy dissipation is slightly less efficient in this case.

Fig 15b presents TL for the three boundary conditions. The reactive liner displays lesser TL, with multiple peaks occurring at specific values of *a*. This reflects the periodic nature of reflection and transmission for the reactive liner, where TL increases at values of *a* corresponding to high reflection and low transmission. The dissipative liners show much higher TL values across the range of *a*, particularly at larger values of *a*. The dissipative condition η=1−i produces the highest TL, with peaks exceeding 50 dB. This indicates that the dissipative liner is highly effective at preventing energy transmission by absorbing most of the incident energy. The condition η=1+i also results in significant TL, though slightly lower than η=1−i. These results demonstrate the effectiveness of dissipative conditions in reducing energy transmission by enhancing absorption.

It is pertinent to mention that the present semi-analytical scheme relies on eigenvalue root-finding for the duct and lined-chamber modes. At some frequencies, roots may cluster (e.g., near cut-on transitions), which can make automated root detection sensitive to initial guesses and may require continuation/root-tracking to avoid missing closely spaced roots. Accuracy also depends on the modal truncation level *N*; increasing *N* improves convergence but increases the cost of matrix assembly and the dense linear solve. In addition, the model assumes linear acoustics and a locally reacting impedance boundary, and we restrict attention to the axisymmetric setting; fully 3D effects, more complex liner physics, and broader validation are left for future work.

## 6 Summary and conclusion

This study investigates the acoustic response of duct systems with elastic membrane components at the interfaces of sound-absorbent lining cavities. A mathematical framework was developed to examine the interaction between duct modes and localized cavity modes, forming a coupled panel-cavity system. The analysis involved solving a boundary value problem where the displacement of circular membrane discs at the interfaces was governed by a second-order differential equation with specific edge conditions. A mode-matching technique was used to determine the undetermined mode amplitudes, ensuring continuity at the interfaces, while the Galerkin formulation was used to characterize the vibrational response of the membrane discs. The findings demonstrate that the proposed configuration effectively enhances acoustic attenuation under various lining conditions and frequency ranges.

The study highlights the role of boundary conditions in shaping acoustic behavior. Soft boundaries reflect most of the incident energy, leading to high transmission loss at low frequencies. In contrast, rigid boundaries allow almost complete energy transmission, resulting in minimal transmission loss. Impedance boundaries exhibit a mixed response, allowing both reflection and transmission depending on the frequency, which leads to moderate transmission loss. These variations emphasize the importance of boundary condition selection in managing energy reflection and transmission within duct systems.

Differences between reactive and dissipative boundary conditions were also examined. Reactive liners (with impedance η=i) primarily reflect and transmit energy with minimal absorption, resulting in moderate TL. Dissipative liners (with impedance η=1+i and η=1−i) absorb more energy, leading to higher TL and reduced transmission power. Among these, the condition η=1−i was found to be the most effective in minimizing transmission, highlighting its potential as an efficient dissipative boundary condition. The impact of chamber geometry on acoustic performance was also explored. Results indicate that reactive liners produce periodic spikes in transmission power and moderate TL. In contrast, dissipative liners effectively reduce both reflected and transmitted power, increasing overall TL. The condition η=1−i consistently demonstrated superior absorption and transmission reduction across different chamber radii, confirming its effectiveness in enhancing acoustic performance.

The study provides valuable insights into the relationship between boundary conditions, chamber geometry, and acoustic performance. The results demonstrate that strategic combinations of boundary conditions and chamber design can significantly improve noise reduction in duct systems, offering practical benefits for industrial and engineering noise control applications.

## Supporting information

S1 Appendix A. Derivation of the dimensionless membrane equations (2.12)–(2.13). (TEX)
